# Editorial: Omics applied to livestock genetics

**DOI:** 10.3389/fgene.2023.1155611

**Published:** 2023-02-16

**Authors:** Lucas Lima Verardo, Luiz F. Brito, Nuno Carolino, Ana Fabrícia Braga Magalhães

**Affiliations:** ^1^ Laboratory of Animal Breeding, Department of Animal Science, Universidade Federal dos Vales do Jequitinhonha e Mucuri, Diamantina, MG, Brazil; ^2^ Department of Animal Sciences, Purdue University, West Lafayette, IN, United States; ^3^ Instituto Nacional Investigação Agraria e Veterinaria (INIAV), Oeiras, Portugal

**Keywords:** data integration, epigenomics, farm animals, genomics, multiomics, proteomics, transcriptomics

Since the first draft of a mammalian genome, a multitude of studies including genomics, transcriptomics, proteomics, epigenomics, and metabolomics datasets aiming to unravel the biological mechanisms influencing phenotypic expression of complex traits have been published (e.g., Portela and Esteller, 2010; Legrain et al., 2011; Costa et al., 2013; Marcobal et al., 2013; Fonseca et al., 2018; Gallagher and Chen-Plotkin, 2018). These “omics” studies have revolutionized the translation of genome to phenome research in the last two decades, including the development of important tools for the livestock sector. There are several projects, initiatives, and databases providing knowledge of genetic variations for the economically, environmentally, and socially important traits in the main livestock species. For instance, the AnimalQTLdb project (Hu et al., 2007) has curated genomic information of a large number of quantitative trait loci (QTL) identified in cattle, pigs, chicken, sheep, and other populations.

In many circumstances, the large-scale datasets generated by livestock “omics” projects have been made publicly available to researchers aiming to generate knowledge and translation tools for improving animal production and sustainability. For instance, the Functional Annotation of Animal Genomes (FAANG) project has generated datasets to decipher the function of genome segments, and it has analyzed samples from approximately 15 species, including pigs, cattle, sheep, and salmon (Giuffra et al., 2019). Moreover, the “omics” approaches can be holistically applied to improve animal breeding strategies based on biology-driven genomic predictions, besides a better understanding of the genomic background of phenotypic variability in livestock systems (Chakraborty et al., 2022).

**FIGURE 1:**
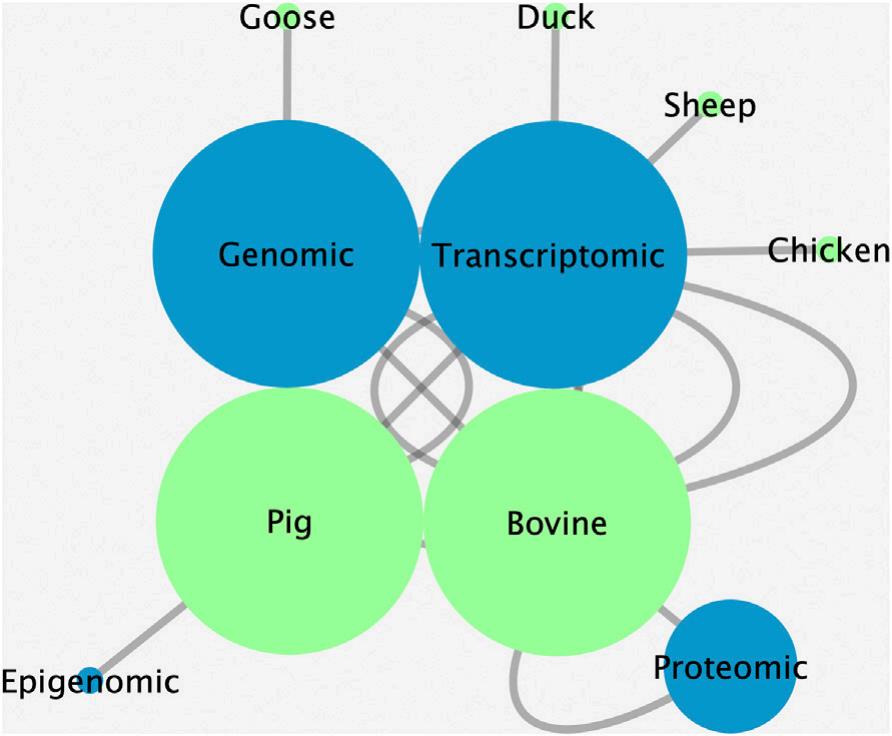
Enrichment analysis highlighting the multi-omics approaches (blue nodes) used and the most livestock species (green nodes) studied in the Research Topic. The size of the nodes corresponds to the Cytoscape network enrichment analysis, as the bigger the nodes, the more used and/or studied were the corresponding nodes.

The Research Topic titled “Omics Applied to Livestock Genetics” presents a collection of the latest findings in the area of livestock genetics based on omics approaches. Studies focusing on food-source animals such as pigs, cattle, ducks, geese, and sheep involving omics data revealed genetic information related to various relevant traits. The two most used approaches were genomics and transcriptomics in cattle and pigs (Figure 1). The results presented provide significant advancements toward understanding farm animal genetics.

In cattle studies, Silva-Vignato et al. combined SNP data from RNA-Seq and a high-density SNP panel to generate a new dataset for performing a genome-wide association analysis (GWAS) for intramuscular fat and backfat thickness in Nellore cattle. Their study revealed genomic regions and regulatory SNPs associated with fat deposition, including transcription factors involved in lipid metabolism-related pathways. Also integrating genomic and transcriptomic data, Liang et al. identified candidate genes for a carcass trait, the weight of *longissimus dorsi* muscle, in Huaxi cattle. After functional analysis of candidate genes and referring to other studies, key genes were suggested to be associated with body development and growth of muscle cells.

A proteomic study integrated with transcriptomic data was presented by Nguyen et al. The authors aimed to elucidate the critical proteins underlying puberty and uncover potential molecular mechanisms from the hypothalamus and pituitary gland of pre-pubertal and post-pubertal Brahman cattle. Their study identified a small number of matched transcripts and protein changes at puberty in each tissue, suggesting the need for multiple omics analyses for a better interpretation of complex biological systems. Moreover, Novais et al. applied factor analysis (FA) and Bayesian network (BN) modeling to integrate proteomic-transcriptomic data and complex traits by latent variables (production, carcass, and meat quality traits) in Nellore cattle. Their framework based on FA and BN generated new hypotheses for molecular research, by integrating different types of data and exploring hidden relationships.

Zhang et al. performed a whole-genome copy number variation detection on Suhuai (SH), Chinese Min Zhu (MZ), and Large White (LW) pigs based on next-generation sequencing data. Copy number variation regions (CNVRs) were annotated and analyzed, with some CNVRs verified by real-time polymerase chain reaction. The authors observed that SH and LW pigs are more closely related and reported annotated genes in CNVRs of each breed. Those genes were related to unique traits in each breed and thus provided important information for the identification of candidate genes for swine breeding. Shi et al. integrated lncRNA-mediated ceRNA network involved in immune regulation in the spleen of Meishan piglets. Their study collected spleen tissues from Meishan piglets at three different ages as a model, and mRNA and lncRNA transcripts were profiled. The interactions between mRNAs and lncRNAs were identified based on weighted gene co-expression network analysis, demonstrating that lncRNAs are a crucial regulatory component in mRNA. The expression of genes related to the immune response of pigs was reported, contributing to a further understanding of the mRNA and lncRNA expression in the spleen of piglets.

Moreover, aiming to explore genomic imprinting, Ahn et al. delineated spatially regulated imprinting of IGF2 transcripts, age-dependent hepatic mono-to biallelic conversion, and reorganization of topologically associating domains at the porcine H19/IGF2 locus for a better translation to human and other animal research. Using a polymorphism-based approach and omics datasets from chromatin immunoprecipitation sequencing (ChIP–seq), whole-genome sequencing, RNA-seq, and Hi-C, regulation of IGF2 during development was analyzed. Their integrative omics analyses of genome, epigenome, and transcriptome provided a comprehensive view of imprinting status at the H19/IGF2 gene cluster.

Gu et al., Gu et al. presented a transcriptome-wide study of embryonic breast muscle development in ducks and chickens, respectively. The authors performed m6A sequencing and miRNA sequencing in the breast muscle of embryos in both species. Several differentially methylated genes and differentially expressed genes were identified. They presented the first characterization of the m6A patterns in the duck transcriptome. Besides, they found that miRNAs, in conjunction with m6A modification, played a key role in the embryonic breast muscle development of Wenchang chickens.

He et al. characterized the microRNA (miRNA) and circular RNA (circRNA) expression profiles in the tail fat of sheep at 6, 18, and 30 months of age. Differentially expressed miRNAs and circRNAs were observed. Functional analysis revealed that miRNA target genes were mainly involved in cellular interactions, while the host genes of circRNAs were associated with lipid and fatty acid metabolism. miRNAs were negatively correlated with circRNAs during sheep tail fat development. Multiple ceRNA regulatory networks dominated by upregulated differentially expressed miRNAs may play a key role in this developmental process. Furthermore, Ni et al. reported whole genome sequencing analyses of a wild swan goose population. They provided a valuable data set for studies on goose genomics. These data may be useful to explore the genetic relationships between the wild swan goose and domestic goose.

In general, we observed that the main production species have been studied through omics approaches. However, multi-omics analyses are still in their infancy and the generation and sharing of multi-omics datasets will be paramount for further advancing research in this field. Functional genomic analyses and high-throughput phenotyping are crucial for providing a clearer picture of the genome-to-phenome paradigm in livestock systems. Moreover, the integration of omics technologies with phenomics into the breeding programs, which was absent from this Research Topic, may help to increase the rates of genetic progress in sustainable breeding programs.
